# The Importance of Epitope Binning for Biological Drug Discovery

**DOI:** 10.2174/1570163810666131124233827

**Published:** 2014-06

**Authors:** Benjamin D. Brooks

**Affiliations:** Wasatch Microfluidics, LLC, 825 North 300 West, Suite C325, Salt Lake City, UT 84103, USA

**Keywords:** Antibody binning, antibody mapping, biotherapeutics drug discovery, drug discovery screening tools, epitope binning, epitope characterization, epitopes, R&D productivity.

## Abstract

The pharmaceutical industry is experiencing comeback sales growth due in large part to the industry’s R&D efforts that center on biologics drug development. To facilitate that effort, tools are being developed for more effective biologic drug development. At the forefront of this effort is epitope characterization, in particular epitope binning, primarily due to the role an epitope plays in drug function. Here we detail the financial advantages of epitope binning including (1) increased R&D productivity due to increased work in process, (2) reduced number of “dead-end”candidates, and (3) increasedability to reengineer antibodies based on the epitope. With the arrival of high throughput biosensors, this manuscript serves as a call to push epitope binning earlier in the biological drug discovery process.

## INTRODUCTION

The emergence of biologics has completely changed the landscape of the pharmaceutical industry. Small molecules still possess profitable short and long term prospects; however, biologics revenues are projected in the near future to outpace small molecules revenues [1]. In a recent study, a large majority of pharma have flat to diminishing revenues from their small-molecule drugs due to patent expirations and generic entry; however, companies that possess revenue from biologics were protected from this revenue erosion [2]. Projections indicate that biologics will continue to capture a rising percentage of the market. This trend reflects both increased use of currently approved biologics and increased approval of new biologics [1, 2]. The trends are telling - total market value of biologics will reach $210 billion by 2016 with seven of the ten medicines being biologics [3]. 

While the principles and general framework for the drug discovery processes are the same for biologics and small molecules many of the tools are different. In addition, the tools for biologics are missing or immature when compared to small molecule drug discovery. In particular, while tools for small molecule drug development including analytical tools for reproducible and high-throughput techniques are mature, they are still in development for biologics drug discovery [4]. Tools that characterize epitopic coverage and affinity are among the most important to develop. In particular, the high throughput drug discovery and development analytical tools need further development. High throughput applications are critical to increasing the R&D productivity for biologics. In the context of new higher throughput biosensors, the opportunity for using epitope binning early in the drug discovery process has emerged.

## EPITOPE BINNING DESCRIPTION

Epitope binning potentially will become a critical, early-stage, screening technique in the biologic drug discovery workflow. In epitope binning antibodies are tested in a pairwise, competition immunoassay [5]. All antibodies in a library are competitively assessed versus all other antibodies in the library to determine which antibodies block the same epitopes on a target antigen. In this manner, a blocking profile for each antibody is determined, and antibodies that possess similarblocking profiles are “binned” together [6]. Engineering mAb that targets a specific functioning epitope on a target antigen (Ag) usually is more important than finding high-affinity, tight-binding mAb, primarily because affinity maturation is a mature and cost effective protein engineering technique [7].

## FINDING THE ‘SWEET SPOT’

In a recent article, Paul *et al.* analyzed the problems of drug discovery in the pharmaceutical industry. Their analysis concluded that the primary threat to the industry is decreasing R&D productivity [8]. Escalating R&D costs combined with the decreasing new drug launches will significantly hamper the innovation necessary to develop the next generation of drugs [8]. The analysis went further in noting that attrition in the late phases of the process to increase R&D productivity (*P*) will require increased numbers in the work in process (WIP) queue while simultaneously reducing cycle time (*CT*) and costs (*C*) (equation 1) [8]. In equation 1, *p*(TS) is the probability of technical success and V is value.

(1) P αWIP×p,TS.×V−CT×C.

**Fig. (1):**
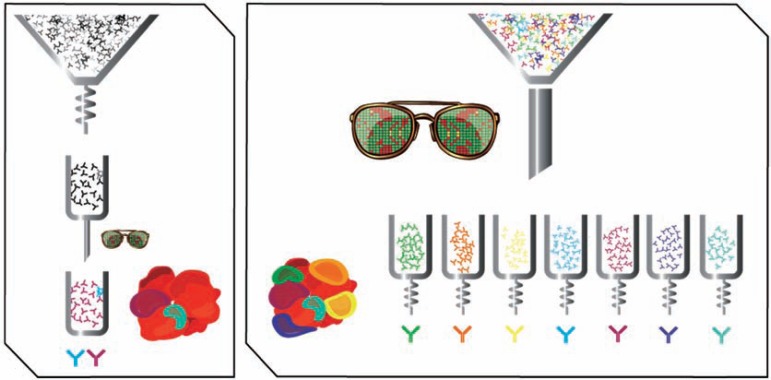
Graphic illustrating the differences in biologic drug discovery process outcomes when epitope binning is used earlier in the process. (**Left panel**) When affinity or other criterion are used as the primary selection tool, one can bias the results to a small number of epitopes. This limits the probability that functional epitopes are represented in the selected panel, or potentially misses candidates with otherwise desirable properties whose affinity could be matured. (**Right panel**) When all candidates are grouped according to epitope first, epitope diversity is maintained and the best performing antibodies in each bin can then be selected.

Epitope binning creates a larger number of WIP entitiesdue to the ability to target different epitopes (Fig. **1**). Intuitively this is done within the workflow of the drug discovery process. A hypothetical case study serves to illustrate the point most effectively. Assume a target has ten epitopes with two epitopes having therapeutic potential. If these epitopes are only the third and eighth most antigenic epitopes, 

it is possible that affinity selection will reach dozens to hundreds of dead ends before a desired functional epitope is discovered. The worst case scenario where these dead ends are discovered in late phase trials is not only possible but also probable. With epitope binning, the top antibodies from each bin are tested, allowing the therapeutically relevant epitopes to be identified more quickly (i.e. less cycle time) and more cost efficiently (i.e. less cost). Most importantly this method could decrease late stage failures resulting in up to a 50% reduction in cost per candidate [8]. The bottom line for epitope binning is that the WIP is increased without increases in costs or cycle time; thus, epitope binning increases the overall probability of success p(TS) in Phase II and III while maintaining large and relevant candidate numbers in the WIP queue. 

**Fig. (2):**
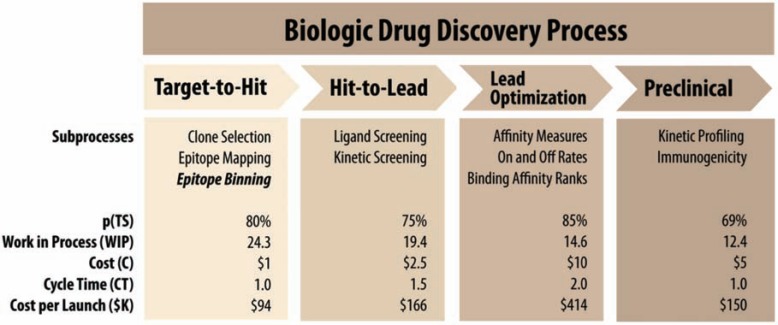
The figure details the distinct phases, processes, and productivity measures of the biologic drug discovery process. We propose that Epitope Binning studies should be conducted at the Target-to-Hit stage of discovery to optimize R&D productivity. The figure is adapted from a model by Paul *et al.*, and is based on a set of industry-appropriate R&D assumptions that define the performance of the R&D process at each stage of development [8]. Parameters for the model include: *p*(TS) is probability of successful transition from one stage to the next, *C* is phase cost for each project, *CT* is cycle time required to progress through each stage of development and the cost of capital, *WIP* is work in process or the number of needed in each stage of development to achieve one new molecular entity (NME) launch.

**Fig. (3):**
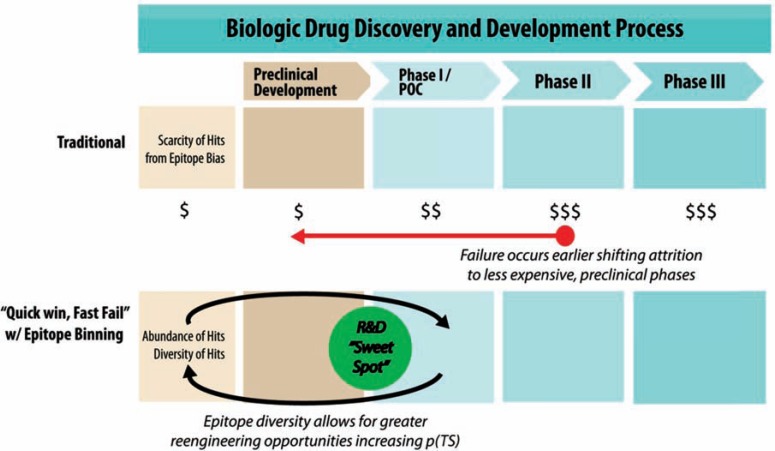
Graphic illustrating the traditional drug development process versus a process with epitope binning referred to as “quick win, fast fail”. In the epitope binning process, failures and technical uncertainty are decreased earlier in the process before expensive Phase II and Phase III trials. This results in a broader more well characterized number of biologics advancing into Phase II and III and those that do advance have a higher probability of success p(TS) and launch (adapted from Paul *et al.*) [8]. Between preclinical development and phase I/ proof-of-concept phase is the R&D “sweet spot.”

The last point in this discussion centers on the “sweet spot”. Epitope binning provides the researcher with important information sooner in the discovery process. As Paul *et al.* noted, reducing the technical uncertainty early in development improves R&D productivity. This approach is referred to as the 'quick win, fast fail' paradigm of drug discovery and development (Fig. **2**). Because information on the epitope and epitope functionality can be discerned sooner, researchers can cycle back and engineer the antibody through processes like affinity maturation earlier creating a feedback in the process (Fig. **3**) for affinity maturation that also reduces costs and decreases cycle time. This feedback fits nicely into the “sweet spot” of drug discovery where resources are optimally used in the early stages of the drug development pipeline (Fig. **2**). This “sweet spot” not only optimizes resources but provides excellent discovery capacity and capability for selection of validated targets. Epitope binning is thus critical to R&D productivity. 

## ELIMINATING THE FUNNEL

Due to limitations in throughput, affinity selection has traditionally been the technique of choice for biologics. Monoclonal antibodies (mAbs) with the highest affinity are then chosen for further characterization. Fig. **1** details the problem with this approach. High affinity antibodies are often limited to a finite number of epitopes and when those epitopes do not impact function in the desired therapeutic role, the end result is a “dead-end” with no effective alternative path, and while some of these dead-ends are identified in early discovery functional test, the dead ends often are discovered in late stages of the drug development process. Epitope binning “eliminates the funnel” shown on the left panel of Fig. **1** and replaces it with multiple pathways to drug development. Moreover, by “eliminating the funnel”, researchers reduce the number of drug candidates, maintain epitope diversity, and reduce epitope bias. Since affinity maturation of antibodies is well understood and relatively easily performed, affinity screening is significantly less desirable financially. Paul *et al.* further detailed that the greatest need for improvement in productivity requires a reduction in Phase II and III attrition. By eliminating these “dead-ends” it drastically decreases attrition rates and thus development costsdue to the high cost of late stage failures (Fig. **2**) [8].

## INCREASING REENGINEERING OPPORTUNITIES

The most significant challenge when engineering biologics remains the fact that both antibodies and epitopes possess properties that could hinder engineering efforts. In epitope binning, even minimal epitope characterization can reveal information for a potential mechanism of action against the target, which allows for a more guided engineering effort for target antigen. Functional antibodies that bind to different epitopes imply different functional therapeutic mechanisms. By generating this information in the target-to-hit and hit-to-lead steps in the drug discovery process, researchers are able to improve lead optimization and improve candidates using affinity maturation (Fig. **2**) [9]. Reengineering allows for reduced dead ends and increases throughput, thereby increase R&D productivity.

## SUMMARY

The greatest challenge of the pharmaceutical industry remains increasing R&D productivity [8]. The escalating costs and decreasing new drug launches are a significant threat to industry survival as the loss of revenues from current drugs will not allow for sufficient innovation of new drugs [8]. Paul *et al.* outlined that the greatest need for improvement in pharma productivity requires a reduction in Phase II and III attrition. The analysis went further in noting that attrition in the late phases of the process will require increasing numbers of drug candidates in the work in process queue while simultaneously reducing cycle time and costs. Using model parameters and sensitivity analyses from Paul *et al.*, we contend that epitope binning can increase the overall *p*(TS) in Phase II and III, increase candidate numbers in the *WIP* queue and provide up to a 50% reduction in cost per drug candidate. Epitope binning is thus critical to maintain the R&D productivity in the industry and should be conducted between the target-to-hit stage of drug discovery and phase I of drug development to optimize R&D productivity. Here we detail that the financial advantages of epitope binning that include (1) increased R&D productivity due to increased work in process, (2) reduced number of “dead-end” candidates, and (3) increased ability to reengineer antibodies based on the epitope.

When Paul *et al.* conducted this study, biologics were just emerging as a significant player in the pharmaceutical industry. Within a few years, however, dozens of new biologics for AIDs, cancer, arthritis, and many difficult to treat diseases will be on the market or in the pipeline for FDA approval. We predict the pessimism and concern expressed by Paul *et al.* will be overshadowed by the increased efficiencies of a new biologic drug discovery and development processes.
